# Associations between disc space narrowing, anterior osteophytes and disability in chronic mechanical low back pain: a cross sectional study

**DOI:** 10.1186/s12891-017-1562-9

**Published:** 2017-05-15

**Authors:** Romain Shanil Perera, Poruwalage Harsha Dissanayake, Upul Senarath, Lalith Sirimevan Wijayaratne, Aranjan Lional Karunanayake, Vajira Harshadeva Weerabaddana Dissanayake

**Affiliations:** 1 Department of Allied Health Sciences, Faculty of Medicine, University of Colombo, 25, Kynsey Road, Colombo 8, Sri Lanka; 2 Department of Anatomy, Faculty of Medical Sciences, University of Sri Jayewardenepura, Gangodawila, Nugegoda, Sri Lanka; 3 Department of Community Medicine, Faculty of Medicine, University of Colombo, 25, Kynsey Road, Colombo 8, Sri Lanka; 4 National Hospital of Sri Lanka, Colombo 10, Sri Lanka; 5 Department of Anatomy, Faculty of Medicine, University of Kelaniya, Annasihena Road, Ragama, Sri Lanka; 6 Department of Anatomy, Faculty of Medicine, University of Colombo, 25, Kynsey Road, Colombo 8, Sri Lanka

**Keywords:** Disability, Disc space narrowing, Anterior osteophytes, Low back pain, Lumbar disc degeneration

## Abstract

**Background:**

Radiographic features of lumbar disc degeneration (LDD) are common findings in patients with chronic mechanical low back pain; however, its role in disability and intensity of pain is debatable. This study aims to investigate the associations of the x-ray features of LDD and lumbar spondylolisthesis with severity of disability and intensity of pain.

**Methods:**

A cross-sectional study was conducted on 439 patients with chronic mechanical low back pain who attended the rheumatology clinic, National Hospital of Sri Lanka, Colombo, from May 2012 to May 2014. Severity of disability was measured using Modified Oswestry Disability Index and intensity of pain was assessed using numeric rating scale (0–100). X-ray features of LDD (disc space narrowing, anterior osteophytes and overall LDD) and spondylolisthesis were assessed in lateral recumbent lumbar x-rays (L1/L2 to L5/S1) and graded by a consultant radiologist blinded to clinical data. Generalised linear model with linear response was used to assess the associations of x-ray features of LDD with severity of disability and intensity of pain adjusting for age, gender, body mass index and pain radiating into legs.

**Results:**

Mean age was 48.99 ± 11.21 and 323 (73.58%) were females. 87 (19.82%) were obese. Mean severity of disability was 30.95 ± 13.67 and mean intensity of pain was 45.50 ± 20.37. 69 (15.72%), 26 (5.92%) and 85 (19.36%) patients had grade 2 disc space narrowing, anterior osteophytes and overall LDD, respectively. 51 (11.62%) patients had lumbar spondylolisthesis. Grade of disc space narrowing and overall LDD were not associated with severity of disability or intensity of pain. The presence of lumbar spondylolisthesis was associated with severity of disability. Female gender and pain radiating into legs were associated with severity of disability and intensity of pain. Advancing age was associated with x-ray features of LDD and lumbar spondylolisthesis.

**Conclusions:**

Lumbar spondylolisthesis is associated with severity of disability in patients with chronic mechanical low back pain. Associations of x-ray features of LDD with severity of disability and intensity of pain are inconclusive. Female gender and pain radiating into legs are significant confounders.

## Background

Disability due to chronic low back pain is one of the leading health care problems in most regions of the world including South Asia [1]. It affects all aspects of life including physical, mental, and social well-being [2]. Disabling chronic low back pain is reported to be a major issue in occupational health in Sri Lanka [3, 4]. Most chronic low back pains are related to mechanical causes including injuries of the musculoskeletal structures of the spine and pathologies associated with lumbar disc degeneration (LDD) [5, 6]. LDD is a common finding in the aging spine and symptoms of chronic mechanical low back pain are not always correlated with the radiological features of LDD. Patients with chronic low back pain receive routine spinal imaging (lumbar x-ray, computed tomography, or magnetic resonance imaging [MRI]) and MRI of lumbar spine has become the popular choice for routine imaging as it gives a direct visualisation of the disc without exposure to the radiation. However MRI is not a cost effective method in routine spinal imaging in developing countries and clinicians in developing countries like Sri Lanka regularly use x-ray lumbar spine as a feasible option for assessing features related to LDD [7].

There are mixed evidence for the association of LDD with chronic mechanical low back pain and disability. Although, routine x-ray of lumbar spine does not affect the outcome of the treatment of uncomplicated acute and subacute low back pain [8], x-ray features related to LDD may benefit the clinical diagnosis and management of chronic low back pain and disability when combined with other factors such as proper history taking, severity of symptoms, surgical risks and costs [8]. Disc space narrowing and anterior osteophytes are the main x-ray features of LDD [9] and are proven to be highly correlated with the morphological stages of LDD [10]. Disc space narrowing is associated with lumbar spinal stenosis, disc herniation and spondylolisthesis which are also related to the pain and disability [11]. Disc space narrowing is associated with the presence of chronic low back pain [9, 12] and intensity of pain [13]. This association becomes stronger with increasing severity of disc space narrowing [12, 13]. Mostly these associations are reported in population based studies and their study samples were limited to middle aged and elderly individuals [9, 12, 13]. There are a limited number of studies which have investigated the association of disc space narrowing with disability [9]. Although anterior osteophyte is the most frequently observed degenerative feature of the aging lumbar spine, it has variably correlated results on its association with intensity of pain [9, 13]. With regard to disability, we could not find enough evidence to prove its association with anterior osteophytes [9, 14]. Both disc space narrowing and anterior osteophytes have been used to determine the grade of overall LDD [15] and high variability exists among the associations between the overall LDD and intensity of pain/disability [14, 16, 17].

Severity of disability/intensity of pain and x-ray features of LDD are further influenced by the effects of age, gender, body mass index (BMI) and the presence of pain radiating into legs. Advancing age increases the susceptibility for severe disability [18]. In most studies females have reported increased intensity of pain and severe disability [19, 20]. In addition obese patients have a higher risk for recurrent disabling low back pain [21]. Furthermore pain radiating into legs is associated with symptomatic disc herniation contributing to severe pain and disability [22]. Age, gender, BMI and the presence of pain radiating into legs may be helpful in predicting the severity of x-ray features of LDD. Advancing age increases the susceptibility for severe degeneration [23, 24]. In addition, there is evidence that males have more degenerative changes compared to females [9], but there are other studies that have given contradicting results [25]. Certain studies have reported that higher BMI has an add-on effect on LDD [26, 27]. However the evidence for associations of gender, BMI and the presence of pain radiating into legs with grade of x-ray features of LDD are inconsistent and need further investigation.

Routine x-ray of lumbar spine is carried out during the management of chronic low back pain in developing countries. Details about age, gender, BMI and the presence of pain radiating into legs are helpful in deciding to prescribe x-ray of lumbar spine as these variables might be useful in predicting the grade of x-ray features of the spine, clinical outcomes and deciding treatment options. Disc space narrowing has significant association with chronic low back pain while anterior osteophytes and LDD have variably correlated results. Most of these studies were population based studies and conducted in middle aged and elderly individuals. There is lack of studies which have assessed the associations of x-ray features of LDD with severity of disability and intensity of pain in patients with chronic mechanical low back pain in clinical settings. There is a wide variation in intensity of pain and disability among patients with chronic mechanical low back pain and patients with severe symptoms require comprehensive care. If there is an association between the grade of x-ray features of LDD, spondylolisthesis and severity of disability and intensity of pain, it would greatly benefit the clinical management with regard to both resource allocation and type of treatment to administer. We hypothesised that the patients with x-ray features of advanced LDD/spondylolisthesis have increased severity of disability and intensity of pain. The objective of our study was to assess the associations of the x-ray features of lumbar disc degeneration and lumbar spondylolisthesis with severity of disability and intensity of pain in patients with chronic mechanical low back pain adjusting for age, gender, BMI and pain radiating into legs. In addition we assessed the associations of x-ray features of LDD with age, gender, BMI and pain radiating into legs.

## Methods

### Study design, setting and participants

A descriptive cross-sectional study was conducted on consecutive patients with chronic mechanical low back pain who attended the rheumatology clinic, National Hospital of Sri Lanka, Colombo, from May 2012 to May 2014. Both male and female patients of Sri Lankan origin with chronic mechanical low back pain aged 20 to 69 years were recruited to the study. Both patients with and without x-ray evidence of LDD and spondylolisthesis were included. Low back pain was defined as pain, muscle tension, or stiffness localized below the costal margin and above the inferior gluteal folds, with or without pain radiating into the leg [19]. Back pain during day time worsening in the latter part of the day due to movements was considered to be due to a mechanical cause [28]. Chronicity was defined as pain on most days of the week for at least three months [2]. Patients with back pain due to inflammatory causes (seronegative spondyloarthropathies, diffuse idiopathic skeletal hyperostosis, rheumatoid arthritis), visceral origin (urinary tract infections, inflammatory pelvic disease), systemic infections affecting spine (spinal tuberculosis), metabolic bone diseases (osteoporosis and osteomalacia), fractures in the vertebral column, past surgeries in the spine, and spinal tumours were excluded. Pregnant females and patients who refuse to participate in the study were also excluded. The study was carried out in accordance with the Declaration of Helsinki and with the approval of the Ethics Review Committee of the Faculty of Medicine, University of Colombo. Patients who fulfilled the inclusion and exclusion criteria were recruited to the study after obtaining written informed consent.

### Clinical evaluation

Demographic (age and gender) and clinical data (intensity of pain, severity of disability, presence of pain radiating into legs, and BMI) were recorded using a pretested interviewer administered questionnaire and clinical examination. The intensity of pain was measured using a 101 (0 to 100) point numeric rating scale. Patients were asked to score the average intensity of pain experienced during the past 7 days out of 100 [29–31]. Disability was assessed using the Modified Oswestry Disability Index (MODI). MODI is a low back pain specific disability questionnaire with ten items which assess pain and its impact on the activities of daily living including personal care, lifting, walking, sitting, standing, sleeping, travelling, social work, home and work duties. Each item has six responses where higher values represent greater disability. Sum of responses was calculated and presented as a percentage [32, 33]. Pain radiating into legs was positive if the pain radiated below the knee of either one or both legs. Height (cm) and weight (kg) of the patients were recorded with light clothing and without shoes to the nearest 0.1 cm and 0.1 kg, respectively, and BMI was calculated (kg/m^2^) [34]. International cut off values were used for categorisation of BMI [35].

### Radiographic evaluation

**Fig. 1:**
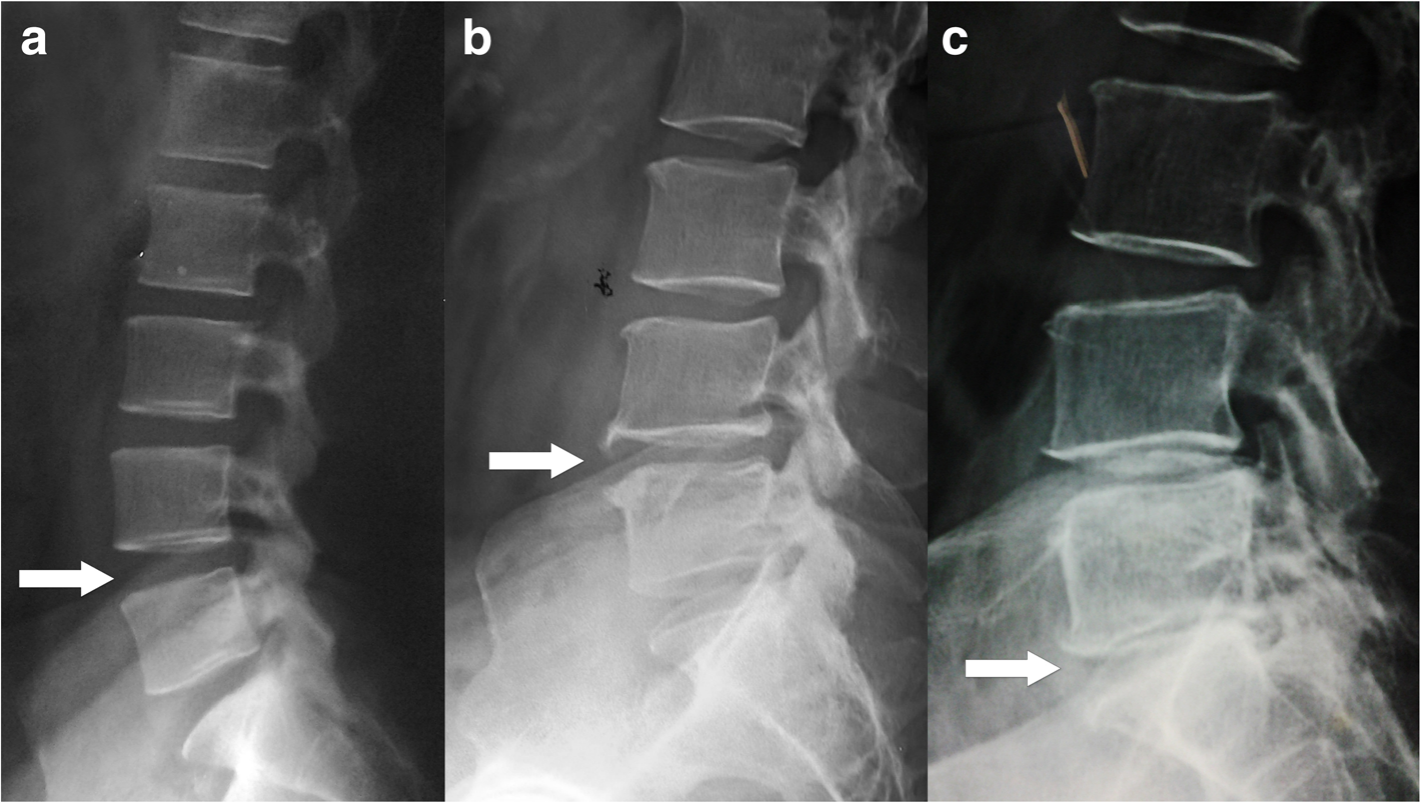
Assesment of the x-ray features of lumbar disc degeneration - lateral x-ray of lumbar spine. Arrows - **a** – no disc space narrowing/anterior osteophyte (grade 0 lumbar disc degeneration), **b** – mild disc space narrowing and small anterior osteophyte, (grade 1 lumbar disc degeneration) **c** – moderate disc space narrowing and small anterior osteophyte (grade 2 lumbar disc degeneration)

Static lateral lumbar x-rays were obtained from all patients. Patients were in lateral recumbent position on the table flexing the knees and hips just enough to achieve comfortable position and a small sandbag was kept between the knees. Midaxillary plane was aligned to the middle of the table and the central x-ray beam was directed perpendicular to the body of the L3 vertebra [36]. Collected lateral lumbar x-rays were evaluated by a consultant radiologist blinded to the clinical details of the patients. The intervertebral disc spaces (L1/L2 to L5/S1) in lateral lumbar x-rays were assessed for the disc space narrowing, anterior osteophytes and lumbar spondylolisthesis. Reduction of the height of the disc space compared to the adjacent normal disc space was defined as the disc space narrowing and presence of bony outgrowths of the vertebral body arising from the borders of superior and inferior surfaces extending anteriorly was defined as anterior osteophyte. Disc space narrowing was graded as follows: grade 0 = none; grade 1 = definite (mild) narrowing; grade 2 = moderate to severe narrowing. Anterior osteophyte was graded as follows: grade 0 = none; grade 1 = small osteophyte and grade 2 = moderate to large osteophyte. Based on these features, overall grading was given for the LDD: grade 0 = normal (grade 0 disc space narrowing and grade 0 anterior osteophyte); grade 1 = grade 1 disc space narrowing and/or grade 1 anterior osteophyte; grade 2 = grade 2 disc space narrowing and/or grade 2 anterior osteophyte (Fig. 1) [37]. End plate sclerosis was not taken into account due to its low interobserver reliability [9, 15]. A particular grade of disc space narrowing/anterior osteophyte/LDD was identified for each of the lumbar levels, and the highest available grade out of the five lumbar levels was used as the final grade for that particular spine. Lumbar spondylolisthesis was defined as presence of displacement of one vertebral body relative to the next most inferior vertebral body and assessed in lateral recumbent lumbar x-ray [11]. However the ability to assess the spondylolisthesis in lateral recumbent lumbar x-ray is limited. Interobserver reproducibility was assessed using a second medical officer who was trained on radiographic evaluation according to the Lane atlas. On random evaluation of 25% of the radiographs were reported by the second medical officer who was blinded to the first reader’s interpretations.

### Statistical analysis

Descriptive statistics were calculated to summarise the sample characteristics. Both univariable and multivariable analyses were carried out. For the univariable analysis, severity of disability and intensity of pain were defined as continuous outcome/dependent variables and independent samples *t*-test was used when there were two categories and Analysis of Variance (ANOVA) was used when there were more than two categories.

Multivariable analysis was performed using different regression models considering the nature of the outcome/dependent variables. Multivariable generalised linear model with linear response was used when the severity of disability and intensity of pain were used as the continuous outcome variables. X-ray features of LDD (disc space narrowing, anterior osteophytes and overall LDD) and presence of lumbar spondylolisthesis were defined as main independent variables/predictor variables and were treated as categorical variables. Separate linear regression models were created for each feature. In each multivariable generalised linear model with linear response, the magnitude of the association was presented as β coefficients with 95% confidence intervals (CI). Multivariable ordinal logistic regression was used when the severity of x-ray features of LDD (disc space narrowing, anterior osteophytes and overall LDD) were used as the ordinal outcome variables (0, 1 and 2). Multivariable logistic regression analysis was used when the presence of lumbar spondylolisthesis (yes/no) was used as a binary outcome variable. Magnitude of the associations was presented as adjusted odds ratios (aOR) with 95% CI in logistic regression models. Age, gender, BMI and presence of pain radiating into legs were defined as confounder variables in all regression models. Age and BMI were treated as continuous variables and gender (male/female) and presence of pain radiating into legs (yes/no) were treated as categorical variables.

Assumptions of ANOVA, independent samples *t*-test and regression models were verified. *P* value < 0.05 was used as the level of significance. Statistical analysis was carried out using SPSS version 17.

## Results

### Characteristics of the participants

Table 1 summarises the characteristics of the study participants. Among 689 patients with chronic mechanical low back pain, 439 patients were recruited according to eligibility criteria. Thirteen patients had missing data for the variable BMI. Mean age ± SD was 48.99 ± 11.21 and 323 (73.58%) were females. BMI ± SD was 26.39 ± 4.65 and 87 (19.82%) were obese. Mean severity of disability was 30.95 ± 13.67 and mean intensity of pain was 45.50 ± 20.37. In addition, 110 (25.10%) patients had pain radiating into legs. With regard to interobserver reproducibility, intra-class correlation coefficient (ICC) of two readers for disc space narrowing was 0.88 (0.82-0.91) and ICC for anterior osteophytes was 0.81 (0.75 – 0.85). Among patients, 176 (40.09%) had disc space narrowing and 201 (45.78%) had anterior osteophytes with 69 (15.72%) and 26 (5.92%) having grade 2 disc space narrowing and grade 2 anterior osteophytes, respectively. LDD was present in 275 (62.64%) and 85 (19.36%) had grade 2 LDD. Lumbar spondylolisthesis was present in 51 (11.62%) patients.

**Table 1:** Summary of sample characteristics

Variable	All *N* = 439 n ((n/N) %)
Age	
20–29 years	26 (5.92)
30–39 years	72 (16.40)
40–49 years	118 (26.88)
50–59 years	141 (32.12)
60–69 years	82 (18.68)
Gender	
Female	323 (73.58)
Male	116 (26.42)
Radiation of pain into legs	
Yes	110 (25.10)
No	329 (74.90)
Body mass index	
Normal (18–24.9 kg/m^2^)	178 (40.55)
Overweight (25–29.9 kg/m^2^)	160 (36.45)
Obese (≥30 kg/m^2^)	87 (19.82)
X-ray features	
Disc space narrowing	
Grade 0	263 (59.91)
Grade 1	107 (24.37)
Grade 2	69 (15.72)
Anterior osteophytes	
Grade 0	238 (54.21)
Grade 1	175 (39.86)
Grade 2	26 (5.92)
Lumbar disc degeneration	
Grade 0	164 (37.36)
Grade 1	190 (43.28)
Grade 2	85 (19.36)
Lumbar spondylolisthesis	
Yes	51 (11.62)
No	388 (88.38)

### Associations of x-ray features of lumbar disc degeneration, spondylolisthesis with severity of disability

There were no significant differences in severity of disability with the severity of disc space narrowing, anterior osteophytes and LDD according to ANOVA and generalised linear models with linear response (Table 2 and 3). Patients with the presence of lumbar spondylolisthesis had significantly severe disability in contrast to the patients without lumbar spondylolisthesis in both univariable and multivariable analysis (Table 2 and 3). Female gender and presence of pain radiating into legs were significantly associated with the severity of disability in all the multivariable generalised linear models (Table 3).

**Table 2:** Means of severity of disability/intensity of pain according to the severity of x-ray features of lumbar disc degeneration and lumbar spondylolisthesis – univariable analysis

Variable	Mean disability ± SD	*p* value	Mean intensity of pain ± SD	*p* value
Disc space narrowing		0.115		0.504
Grade 0	30.48 ± 12.92		44.63 ± 19.58	
Grade 1	30.10 ± 13.84		47.31 ± 21.69	
Grade 2	34.07 ± 15.81		46.01 ± 21.33	
Anterior osteophytes		0.076		0.200
Grade 0	30.05 ± 13.61		44.10 ± 20.20	
Grade 1	32.65 ± 14.15		47.64 ± 20.73	
Grade 2	27.79 ± 9.17		43.87 ± 18.93	
Lumbar disc degeneration		0.207		0.534
Grade 0	29.75 ± 12.85		44.13 ± 19.05	
Grade 1	31.07 ± 13.79		46.11 ± 21.32	
Grade 2	32.98 ± 14.79		46.79 ± 20.77	
Lumbar spondylolisthesis		0.001*		0.289
Yes	36.65 ± 13.58		48.34 ± 19.97	
No	30.20 ± 13.52		45.13 ± 20.42	

* - *p* value < 0

*SD* standard deviation

**Table 3:** Associations of x-ray features of lumbar disc degeneration and spondylolisthesis with severity of disability and intensity of pain – multivariable generalised linear models with linear response

	Severity of disability		Intensity of pain	
Variables	β coefficient (95% confidence itervals)	p value	β coefficient (95% confidence intervals)	*p* value
Regression model 1				
Intercept	22.945 (14.589 – 31.301)	<0.001*	47.375 (34.766 – 59.984)	<0.001*
Severity of disc space narrowing				
Grade 0	0.000		0.000	
Grade 1	-0.311 (-3.301 – 2.679)	0.839	3.896 (-0.616 – 8.408)	0.091
Grade 2	2.751 (-0.934 – 6.437)	0.143	2.007 (-3.555 – 7.569)	0.479
Age (years)	-0.014 (-0.133 – 0.106)	0.824	-0.173 (-0.352 – 0.007)	0.060
Gender				
Male	0.000		0.000	
Female	7.369 (4.446 – 10.293)	<0.001*	4.899 (0.488 – 9.311)	0.030*
Body mass index (kg/m^2^)	0.042 (-0.236 – 0.320)	0.765	-0.049 (-0.468 – 0.370)	0.819
Pain radiating into legs				
No	0.000		0.000	
Yes	6.989 (4.174 – 9.803)	<0.001*	12.262 (8.015 – 16.508)	<0.001*
Regression model 2				
Intercept	22.049 (13.736 – 30.362)	<0.001*	49.630 (37.099 – 62.161)	<0.001*
Severity of anterior osteophyte				
Grade 0	0.000		0.000	
Grade 1	2.136 (-0.565 – 4.838)	0.121	4.836 (0.764 – 8.908)	0.020*
Grade 2	-2.309 (-7.727 – 3.108)	0.403	2.931 (-5.235 – 11.097)	0.482
Age	0.001 (-0.118 – 0.120)	0.989	-0.200 (-0.379 – -0.021)	0.029*
Gender				
Male	0.000		0.000	
Female	7.374 (4.448 – 10.301)	<0.001*	5.507 (1.096 – 9.918)	0.014*
Body mass index (kg/m^2^)	0.037 (-0.241 – 0.315)	0.794	-0.130 (-0.550 – 0.289)	0.543
Pain radiating into legs				
No	0.000		0.000	
Yes	6.889 (4.076 – 9.702)	<0.001*	12.045 (7.805 – 16.285)	<0.001*
Regression model 3				
Intercept	22.710 (14.255 – 31.164)	<0.001*	49.254 (36.523 – 61.985)	<0.001*
Severity of lumbar disc degeneration				
Grade 0	0.000		0.000	
Grade 1	0.976 (-1.885 – 3.838)	0.252	3.642 (-0.688 – 7.951)	0.098
Grade 2	2.243 (-1.599 – 6.086)	0.504	4.559 (-1.227 – 10.345)	0.122
Age	-0.020 (-0.144 – 0.105)	0.758	-0.207 (-0.394 - -0.020)	0.030*
Gender				
Male	0.000		0.000	
Female	7.320 (4.391 – 10.249)	<0.001*	5.240 (0.829 – 9.651)	0.020*
Body mass index (kg/m^2^)	0.045 (-0.234 – 0.324)	0.753	-0.111 (-0.531 – 0.310)	0.606
Pain radiating into legs				
No	0.000		0.000	
Yes	7.009 (4.191 – 9.828)	<0.001*	12.167 (7.923 – 16.412)	<0.001*
Regression model 4				
Intercept	23.304 (15.086 – 31.523)	<0.001*	48.248 (35.749 – 60.748)	<0.001*
Presence of lumbar spondylolisthesis				
No	0.000		0.000	
Yes	5.670 (1.684 – 9.656)	0.005*	3.549 (-2.514 – 9.612)	0.251
Age	-0.040 (-0.156 – 0.076)	0.501	-0.168 (-0.345 – 0.009)	0.063
Gender				
Male	0.000		0.000	
Female	7.011 (4.114 – 9.908)	<0.001*	4.824 (0.418 – 9.231)	0.032*
Body mass index (kg/m^2^)	0.078 (-0.197 – 0.353)	0.578	-0.055 (-0.473 – 0.363)	0.795
Pain radiating into legs				
No	0.000		0.000	
Yes	6.820 (4.019 – 9.621)	<0.001*	12.128 (7.868 – 16.388)	<0.001*

Main predictor (independent) variables were analysed as follows:

Regression model 1: severity of disc space narrowing (grade 0, 1 and 2)

Regression model 2: severity of anterior osteophytes (grade 0, 1 and 2)

Regression model 3: severity of lumbar disc degeneration (grade 0, 1 and 2)

Regression model 4: presence of lumbar spondylolisthesis (yes/no)

* - *p* value < 0.05

### Associations of x-ray features of lumbar disc degeneration, spondylolisthesis with intensity of pain

Disc space narrowing and LDD were not associated with intensity of pain in either univariable or multivariable regression analyses (Table 2 and 3). However patients with grade 1 anterior osteophytes had significantly higher intensity of pain compared to the patients with grade 0 anterior osteophytes. The presence of lumbar spondylolisthesis was not associated with the intensity of pain. Female gender and pain radiating into legs were associated with the intensity of pain in all multivariable generalised linear models (Table 3). In addition increasing age was associated with two linear regression models involving anterior osteophytes and LDD (Table 3).

### Associations of age, gender, BMI and presence of pain radiating into legs with x-ray features of lumbar disc degeneration and spondylolisthesis

The presence of grade 2 disc space narrowing was reported from 20 – 29 years age group, but presence of grade 2 anterior osteophytes was reported from 40 – 49 years age group. Furthermore the presence of lumbar spondylolisthesis was reported from 30 – 39 years age group. Advancing age was strongly associated with the severity of disc space narrowing, anterior osteophytes, LDD (Table 4) and presence of lumbar spondylolisthesis (aOR 1.15; 95% CI: 1.1 – 1.21) after adjusting for gender, BMI and pain radiating into legs. Male gender was associated with the severity of anterior osteophytes, but was not associated with disc space narrowing, overall LDD and lumbar spondylolisthesis. Furthermore, BMI was significantly associated with grades of anterior osteophytes and LDD.

**Table 4:** Associations of age, gender and BMI with x-ray features of lumbar disc degeneration – multivariable ordinal logistic regression model

	Disc space narrowing			Anterior osteophytes			Disc degeneration		
Variable	Parameter estimates (β)	aOR (95% CI)	*p* value	Parameter estimates (β)	aOR (95% CI)	*p* value	Parameter estimates (β)	aOR (95% CI)	*p* value
Age	0.074	1.077 (1.056 – 1.100)	<0.001*	0.075	1.078 (1.056–1.100)	<0.001*	0.090	1.094 (1.072–1.115)	<0.001*
BMI	0.023	1.023 (0.980–1.069)	0.301	0.058	1.059 (1.013–1.108)	0.012*	0.060	1.062 (1.018–1.108)	0.005*
Gender									
Male	-0.245	1.277 (0.792 – 2.059)	0.316	0.517	1.676 (1.042–2.696)	0.033*	0.371	1.450 (0.928–2.265)	0.103
Female	0.000			0.000			0.000		
Pain radiating into legs									
No	–0.161	0.851 (0.542 – 1.335)	0.483	-0.118	0.888 (0.566–1.394)	0.606	-0.168	0.845 (0.551–1.296)	0.441
Yes	0.000	1.175 (0.749–1.844)		0.000			0.000		

* - *p* value <0.05

aOR adjusted odds ratios, CI confidence interval

## Discussion

In this study we assessed the associations of x-ray features of LDD and lumbar spondylolisthesis with severity of disability and intensity of pain in patients with chronic mechanical low back pain adjusting for age, gender, BMI and pain radiating into legs. In addition we assessed the associations of x-ray features of LDD with age, gender, BMI and presence of pain radiating into legs. We found that, the associations of x-ray features of LDD with severity of disability or intensity of pain (except anterior osteophytes) were inconclusive. The presence of lumbar spondylolisthesis was associated with increased severity of disability. However the Female gender and presence of pain radiating into legs were associated with increased severity of disability and intensity of pain. Furthermore, x-ray features of LDD and lumbar spondylolisthesis were strongly associated with advancing age.

Lumbar intervertebral discs are fibrocartilage pads between adjacent lumbar vertebral bodies which distribute compressive loading evenly on to the vertebral bodies. Intervertebral discs contribute to spinal stability along with the apophyseal joints and supported by surrounding muscles and ligaments [38]. With LDD the normal architecture of the disc is disrupted leading to abnormal biomechanical force distribution which may cause severe and disabling low back pain. With degeneration, the height of the disc can be reduced due to inward or outward herniation of the disc material and is visible as disc space narrowing in x-ray lumbar spine. This results in abnormal load distribution to the surrounding structures and lead to segmental instability and spondylolisthesis. Formation of osteophytes is a compensatory mechanism to distribute increasing axial forces of spine on a larger articulating surface to prevent spinal instability [11]. Although x-ray features of LDD are not correlated with the outcome of the treatment, they can give important details for managing chronic mechanical low back pain especially in the presence of severe symptoms [39].

Disc space narrowing is used as a surrogate variable for LDD and many studies found positive association with the presence of chronic low back pain in population based studies [9, 15, 24]. However, studies done in clinical settings did not find significant association between disc space narrowing and intensity of pain [17]. Similarly in our study disc space narrowing was not associated with intensity of pain. There are limited cross sectional clinical studies which have assessed the association of LDD with disability. A study on 172 consecutive patients with chronic low back pain in United Kingdom did not find significant association between LDD (based on x-ray findings) and disability [15]. Authors of the previous study did not assess the association of features of LDD separately as disc space narrowing and anterior osteophytes, but rather assessed the overall LDD. According to our univariable and multivariable analyses disc space narrowing was not associated with disability, but gender and presence of pain radiating into legs had significant association with disability.

Comparatively, the association between anterior osteophytes and chronic low back pain is largely considered as not significant, unless there is a higher grade of anterior osteophytes [9, 24]. As mentioned previously we could not find cross sectional clinical studies which have assessed the associations between anterior osteophytes and disability. Higher grades of anterior osteophytes are frequently seen in elderly individuals (above 65 years). Our sample was restricted to patients below 70 years and there were only 26 patients with grade 2 anterior osteophytes. In our results, grade of anterior osteophytes was not associated with the severity of disability. However the patients with grade 1 anterior osteophytes had higher intensity of pain in contrast to patients with grade 0 anterior osteophytes. The overall association between the grades of anterior osteophytes and intensity of pain was inconsistent as there was no significant association between intensity of pain and grade 2 anterior osteophytes in contrast to grade 0 anterior osteophytes.

In most studies, overall LDD poorly correlated with clinical symptoms including severity of disability and intensity of pain [14, 40]. In our results we could not find a significant association between LDD and severity of disability or LDD and intensity of pain, which agree with the findings of the previous evidence [15]. Most radiographic scoring systems including the Lane atlas have used disc space narrowing or anterior osteophytes or both features to determine the overall LDD. Accordingly, either higher grades of disc space narrowing or higher grades of anterior osteophytes could determine higher grades of LDD. Although grade 1 anterior osteophytes was associated with intensity of pain (in contrast to patients with grade 0 anterior osteophytes), we could not find a significant association between overall LDD and intensity of pain. The strength of the association might have become further attenuated when both features (disc space narrowing and anterior osteophytes) were considered in overall LDD.

Degenerative lumbar spondylolisthesis is related to LDD and degenerative changes of the apophyseal joints [11]. In our results presence of lumbar spondylolisthesis was associated with the increasing grade of disc space narrowing and overall LDD, and was more frequent at the L4–L5 level. The presence of lumbar spondylolisthesis was associated with increased severity of disability in our study, but it was not associated with the intensity of pain. Narrowing of the disc space is associated with advanced LDD, annular tears and disc herniation, but these features do not always correlate well with the intensity of pain. Furthermore it can adversely affect the biomechanical stability of the lumbar spine which will increase the strain on apophyseal joints and surrounding structures where the combined effects can reduce the flexibility and stability of the spine leading to severe disability [11].

X-ray features of LDD are age related [9, 24] and our study results are compatible with the previous evidence. Interestingly, disc space narrowing was seen from an early age (20 –39 years), but anterior osteophytes was seen from the middle age group (40 –49 years) onwards. Although previous studies have found significantly higher degenerative features in males [9], we found positive association only with anterior osteophytes. Furthermore, increasing BMI was associated with increasing grade of anterior osteophytes and LDD which was compatible with previous findings [26, 27].

There is evidence that females are more susceptible to higher intensity of pain and disability and our results were compatible with the existing evidence. Finding reasons for this is beyond the objectives of our study, however, certain studies have suggested that females have higher sensitization to pain, higher chance of reporting of pain and differences in response to analgesics [41–43]. The presence of pain radiating into legs was strongly associated with severity of disability and intensity of pain. Pain radiating into legs is associated with symptomatic disc herniation, annular tears and nerve impingement which can cause severe disability and pain [22]. These two variables have strong confounding effect on the associations between x-ray features of LDD/lumbar spondylolisthesis and severity of disability/intensity of pain.

As there are less certain radiographic recommendations for uncomplicated chronic mechanical low back pain [44], regular radiographic assessment (x-ray lumbar spine) are taken into account during the decision making on different treatment options. In management of chronic mechanical low back pain weight training is a viable option in patients with mild LDD, but presence of moderate to severe features of LDD make this option unjustifiable. Furthermore, patients with lumbar spondylolisthesis may require specific flexion strengthening exercises during the management to reduce the pain and disability [45]. The presence of lumbar spondylolisthesis, female gender and pain radiating into legs increased the severity of disability in our patients and these features might provide helpful information when assessing the severity of disability and management decision on type of treatment to administer.

There are a few limitations in the study. Our study is cross-sectional and was conducted in a specific group of patients with chronic mechanical low back pain at a single centre. We have not assessed the other associated factors with disability and pain such as depression, anxiety and fear avoidance. In addition we have not assessed the dynamic stability of the lumbar spine which could have contributed to the severity of disability and intensity of pain. X-ray lumbar spine cannot visualise the intervertebral disc directly. There may be increased risk of type 1 error due to multiple comparisons and it may affect the significance of the findings.

## Conclusions

This study shows that the predictive ability of x-ray features of LDD for severity of disability and intensity of pain is weak among the patients with chronic mechanical low back pain. However the presence of lumbar spondylolisthesis is associated with severe disability. Female gender and the presence of pain radiating into legs are associated with increased severity of disability and intensity of pain, hence acting as strong confounders. Advancing age is associated with x-ray features of advanced LDD including spondylolisthesis. The presence of lumbar spondylolisthesis, gender and pain radiating into legs are good predictive factors of severe disability and higher intensity of pain which may facilitate the decision making process in management of chronic mechanical low back pain.

## Abbreviations

aOR: Adjusted odds ratios
BMI: Body mass index
CI: Confidence intervals
ICC: Intra-class correlation coefficient
LDD: Lumbar intervertebral disc degeneration
MODI: Modified oswestry disability index
SD: Standard deviation
